# Evaluating molecular representations in machine learning models for drug response prediction and interpretability

**DOI:** 10.1515/jib-2022-0006

**Published:** 2022-08-26

**Authors:** Delora Baptista, João Correia, Bruno Pereira, Miguel Rocha

**Affiliations:** Centre of Biological Engineering, University of Minho, Campus of Gualtar, Braga, Portugal

**Keywords:** cancer, deep learning, drug sensitivity, learned representations, molecular fingerprints

## Abstract

Machine learning (ML) is increasingly being used to guide drug discovery processes. When applying ML approaches to chemical datasets, molecular descriptors and fingerprints are typically used to represent compounds as numerical vectors. However, in recent years, end-to-end deep learning (DL) methods that can learn feature representations directly from line notations or molecular graphs have been proposed as alternatives to using precomputed features. This study set out to investigate which compound representation methods are the most suitable for drug sensitivity prediction in cancer cell lines. Twelve different representations were benchmarked on 5 compound screening datasets, using DeepMol, a new chemoinformatics package developed by our research group, to perform these analyses. The results of this study show that the predictive performance of end-to-end DL models is comparable to, and at times surpasses, that of models trained on molecular fingerprints, even when less training data is available. This study also found that combining several compound representation methods into an ensemble can improve performance. Finally, we show that a *post hoc* feature attribution method can boost the explainability of the DL models.

## 1 Introduction

ML has been widely used in the pharmaceutical industry for rational drug discovery. Quantitative structure-activity relationship (QSAR) models, for example, typically use ML algorithms to learn the relationship between the structures or properties of compounds and their biological activity. ML can help guide the discovery process by identifying the most promising drug candidates before experimental work is carried out. The development of predictive models of drug response in cancer is a particularly important application of ML in this field [1, 2].

**Figure 1:**
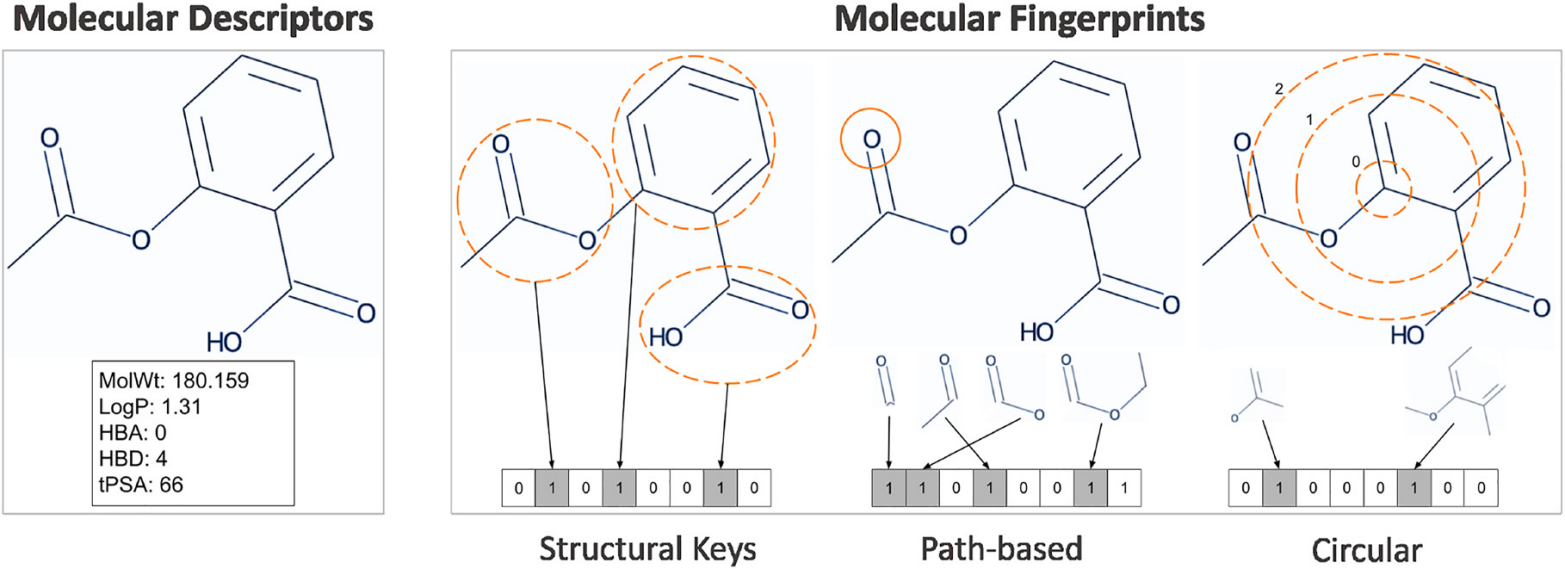
Traditional representations of compounds for machine learning tasks.

The first step in an ML workflow for drug discovery is usually the calculation of molecular descriptors or fingerprints (Figure 1). Molecular descriptors are the experimental or theoretical physicochemical properties of a compound. Molecular fingerprints encode molecules as bit or count vectors. The information that is encoded depends on the type of fingerprint. Substructure key-based fingerprints set the bits of the bit vector to one or zero depending on the presence or absence in the compound of certain substructures or features from a list of predefined structural keys [3]. Path-based fingerprints enumerate the different linear paths between atoms in a molecule to determine which types of fragments are present. Circular fingerprints describe the surrounding environment of each atom in the molecule up to a predefined radius [3].

**Figure 2:**
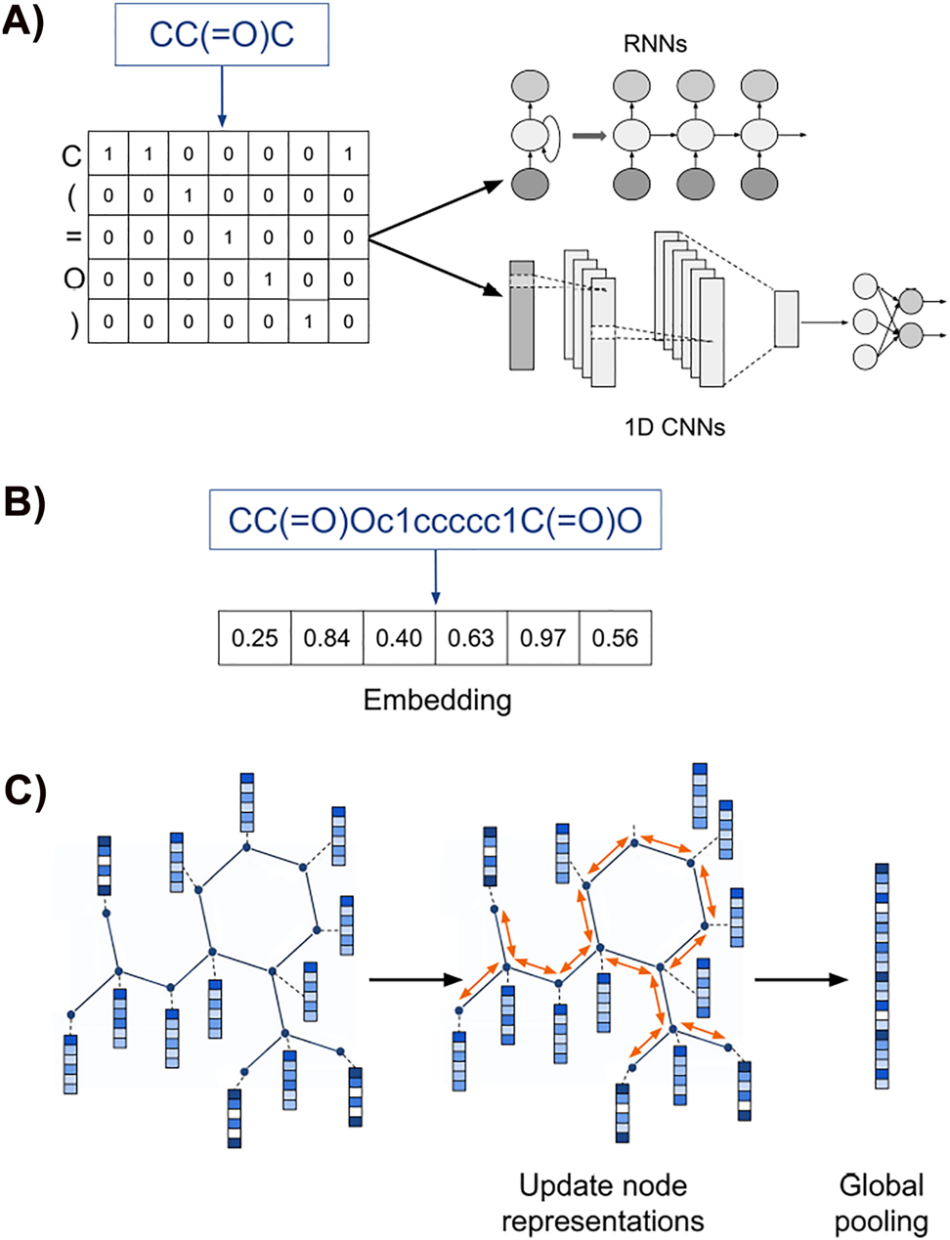
Learned representations of compounds. (A) Representations can be learned directly from SMILES strings using DL architectures such as RNNs or 1D CNNs. (B) Natural language processing-inspired methods can be used to generate continuous embeddings of molecules. (C) GNNs can be used to learn representations directly from molecular graphs.

The use of end-to-end DL approaches (Figure 2) that can learn relevant features directly from raw input data may eliminate the need for precomputed descriptors and fingerprints. Graph neural networks (GNNs) can learn directly from molecular graphs [4, 5]. Certain types of DL algorithms, such as recurrent neural networks (RNNs) and 1D convolutional neural networks (CNNs), are able to learn from line notations like Simplified Molecular-Input Line-Entry System (SMILES) strings, and DL-based natural language processing methods can be applied to line notations to create continuous embeddings of molecules [6].

Several recently published benchmarking studies have analyzed whether learned representations of compounds can outperform molecular descriptors and fingerprints. The authors of one such study compared different representation methods across several drug target prediction tasks and reached the conclusion that end-to-end DL methods tended to perform worse than DL models trained using precomputed chemical features [7]. Another study benchmarked a variety of molecular descriptor-based ML models and GNN models on several molecular property prediction datasets, and also concluded that descriptor-based models usually outperformed the GNN models [8]. A different research group found that GNNs performed better than fully-connected neural networks (FCNNs) trained with molecular fingerprints on most of the benchmarking tasks considered in the study [9]. Another recent study evaluated the performance of several molecular fingerprints and DL-based representations on drug combination sensitivity and synergy prediction tasks using a large drug combination dataset. The authors found that several DL-based representations outperformed traditional fingerprints, but they also noted that the differences in performance between traditional and learned representations were small [10]. A large-scale benchmarking study using MoleculeNet datasets concluded that learned representations perform worse when training data is scarce or very imbalanced [11], and several other studies have also observed that traditional fingerprints tend to outperform learned representations in low data scenarios [12, 13]. Therefore, the most suitable representation for a given prediction problem probably depends on the type of problem itself, as well as other factors such as dataset size, making it essential to evaluate this for each specific application.

In this study, a variety of molecular representation methods and DL algorithms were benchmarked to determine which representations are the most suitable for predicting drug sensitivity in cancer cell lines. A new Python package, developed in-house, was used to perform this analysis. All of the datasets and scripts used in this study are available online at https://github.com/BioSystemsUM/DeepMol/tree/pacbb21/pacbb21_paper.

## 2 Methods

### 2.1 Datasets

A selection of DL models were benchmarked on several human cancer cell line drug screening datasets (Table 1). Single-cell line datasets were used so that it would be possible to study the effect of different compound representations without having to take cell line features into account.

**Table 1:** Details on the datasets used in this study.

Dataset	Compounds	Output variable	Task type
NCI 1	3466	Sensitive/not sensitive	Classification
NCI 109	3431	Sensitive/not sensitive	Classification
PC-3	4294	−log(IC_50_)	Regression
CCRF-CEM	3047	−log(IC_50_)	Regression
A549/ATCC	20,730	−log(GI_50_)	Regression

The NCI 1 and NCI 109 human tumor cell line growth inhibition datasets were used to develop binary classification models. These datasets were chosen because they have been widely used in the literature to validate graph classification algorithms. In this study, balanced versions of these datasets [14] were used (available from https://github.com/shiruipan/graph_datasets). In each dataset, the output variable indicates whether a given compound is active or inactive in a specific cell line.

To develop and evaluate regression models, two single-cell line cytotoxicity datasets (PC-3 and CCRF-CEM) from a recent drug sensitivity prediction study [15] were used. The authors of this study obtained the original datasets from ChEMBL [16], performed some filtering and data cleaning steps, and transformed the original half maximal inhibitory concentration (IC_50_) values into −log(IC_50_) values (pIC_50_). These pIC_50_ values were used as the output variable for the regression models.

The previously mentioned datasets are all relatively small, each comprised of less than 5000 compounds. However, these smaller datasets were preferred because it was assumed that they would more closely reflect the behavior of the compound representation methods when used in drug sensitivity prediction models trained on publicly available anti-cancer screening datasets, which usually have data for even fewer compounds. Indeed, the original version of the popular Genomics of Drug Sensitivity in Cancer (GDSC) resource [17], for example, provides access to a dataset (GDSC1) containing screening data for only 367 compounds, while the Cancer Therapeutics Response Portal (CTRPv2)v2 [18] dataset has data for only 481 compounds.

Nevertheless, a larger dataset derived from the National Cancer Institute 60 Human Cancer Cell Line Screen (NCI-60) dataset was also used for benchmarking. A single cell line, A549/ATCC, was selected from this dataset. After removing low quality experiments, compounds without sensitivity data, and compounds that could not be mapped to SMILES strings using the files provided by the Developmental Therapeutics Program (DTP), the final dataset contained 20,730 compounds. Sensitivity was measured as −log(GI_50_ (half maximal growth inhibition concentration)) in this dataset.

Prior to modeling, all SMILES strings were preprocessed using the ChEMBL Structure Pipeline [19].

### 2.2 Models

#### 2.2.1 Pre-computed features

Six different types of molecular fingerprints were evaluated in this work: extended connectivity fingerprint (ECFP) (ECFP4 and ECFP6), Molecular ACCess System (MACCS) keys, atom pair fingerprints (AtomPair), RDKit fingerprints (RDKitFP) and RDKit layered fingerprints (LayeredFP). ECFP fingerprints [20] are a popular circular fingerprint based on the Morgan algorithm [21]. ECFP4 fingerprints use a radius of 2 to define the circular neighborhood surrounding each atom, while ECFP6 fingerprints use a radius of 3. MACCS is a type of substructure key-based fingerprint which uses 166 predefined keys [22]. The AtomPair fingerprint is a topological fingerprint based on determining the shortest distance between all pairs of atoms within a molecule [23]. The RDKitFP is another topological fingerprint that was developed by the RDKit [24] project. The algorithm finds all subgraphs in a molecule containing a number of bonds within a predefined range, hashes the subgraphs, and then uses these hashes to generate a bit vector of fixed length. The LayeredFP [24] uses the same algorithm as the RDKitFP to identify subgraphs, but different bits are set in the final fingerprint based on different “layers” (different atom and bond type definitions).

Compound structures were encoded as bit vectors using each fingerprinting algorithm and the resulting fingerprints were used as inputs to FCNNs. With the exception of MACCS fingerprints, which have a fixed length, the size of all fingerprints was limited to 1024 bits.

#### 2.2.2 Mol2vec embeddings

Mol2vec is an unsupervised method that generates continuous vectors representing molecules using the Word2vec word embedding algorithm [6]. Each molecule is considered a “sentence” and molecular substructures (calculated using the Morgan algorithm) are considered “words”. In this work, a pre-trained Mol2vec model was used to generate 300-dimensional embeddings for the molecules in each dataset, which were fed into FCNNs.

#### 2.2.3 TextCNN

TextCNN is a 1D CNN that was originally developed for sentence classification [25]. A modified version of this algorithm (implemented in DeepChem [26]), which uses tokenized and one-hot encoded SMILES strings as inputs instead of words, was used in this study. It applies several 1D convolutional filters, followed by a max-over-time pooling operation, which summarizes each filter using its maximum value. These learned features are then fed into fully-connected layers to predict the output.

#### 2.2.4 Graph neural networks

The structure of a chemical compound can be represented as a molecular graph, where nodes are atoms and edges represent bonds. Conventional neural network architectures, such as FCNNs or CNNs, are unable to learn directly from this type of data.

GNNs generalize deep neural networks to graph-structured data. Inputs to a GNN are usually node features (e.g. atom type) and adjacency matrices encoding the structure of the graph, and sometimes can also include edge features as well. The node and edge features are used to initialize the graph. In general, GNNs apply learnable functions to update the node-level representations, progressively incorporating information about the neighborhood of a node into its representation. After several rounds of updates, a pooling operation can be used to obtain a graph-level (molecular-level) representation. The graph-level representations can then be fed into fully-connected layers to predict a given output.

In this work, four different GNN algorithms were benchmarked: neural fingerprints (GraphConv) [4], graph convolutional network (GCN) [27], graph attention network (GAT) [28] and the AttentiveFP algorithm [5].

### 2.3 Model training and evaluation

**Figure 3:**
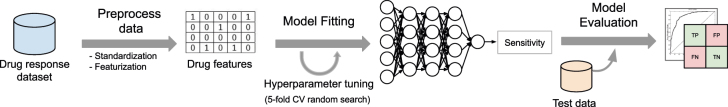
General data preprocessing and machine learning steps followed in this work.

The modeling workflow that was followed in this study is shown in Figure 3.

Each dataset was split into a training set (70%) and a test set (30%). All models were trained and evaluated using the same splits for each dataset.

All models were trained for 100 epochs with a batch size of 256 samples, and used the Adam [29] optimization algorithm. Cross-entropy loss was used as the loss function for all classification models, while the mean squared error was used for regression models. Other model-specific hyperparameters were tuned using a 5-fold cross-validated randomized search, in which 30 different hyperparameter combinations were tested. These included the number of hidden layers and hidden units and the use of regularization methods, such as L2 weight regularization and dropout [30], among others. The best model that was found for each algorithm was then refit on the entire training set and evaluated on the held-out test set. Additional details on the models (including the full search space and the best hyperparameters found for each type of model) are available online at https://github.com/BioSystemsUM/DeepMol/blob/pacbb21/pacbb21_paper/supplementary_material.pdf.

### 2.4 Model ensembling

Besides evaluating individual models, simple voting ensembles were also built to determine if combining predictions from multiple models could further improve performance. For classification tasks, majority voting ensembles were built, where the final prediction is the label predicted by the majority of the individual classifiers. For regression tasks, the ensembles simply averaged the individual predictions to obtain the final prediction. The hyperparameter values that were used for each individual model were the optimal values that had been previously tuned using randomized search.

### 2.5 Feature importance

Feature importance was determined using the Deep SHapley Additive exPlanations (SHAP) method implemented in the SHAP Python package (version 0.39.0) [31]. Deep SHAP approximates SHAP values for DL models by using a modified version of the DeepLIFT [32] algorithm.

### 2.6 DeepMol

All preprocessing, featurization and modeling steps were implemented using DeepMol, a newly developed chemoinformatics package from our host group. It is a python-based machine and deep learning framework for drug discovery, offering a variety of functionalities that enable a smoother approach to many drug discovery and chemoinformatics problems. This framework uses Tensorflow [33], Keras [34], Scikit-learn [35] and DeepChem [26] to either build custom ML and DL models or make use of pre-built models. It also uses the RDKit [24] framework to perform operations on molecular data.

Regarding compound standardization, it allows users to use the ChEMBL Structure Pipeline [19] or apply custom standardization steps using RDKit [24] standardization methods. Some of these steps include standardization of some non-standard valence states, molecule sanitization, charge neutralization, stereochemistry removal, the removal of smaller fragments, kekulization, among others.

The package also offers several featurization methods including molecular fingerprints, molecular embeddings and graph-based featurizers.

In summary, the package offers a complete workflow to perform machine and deep learning tasks for molecules represented as SMILES strings. It has modules that perform standard tasks, such as loading and standardizing data, computing molecular features, performing feature selection and data splitting. It also provides methods to deal with unbalanced datasets and to do unsupervised exploration of the data. This way, DeepMol provides a common platform to treat the data and build, train, optimize and evaluate ML and DL models using different ML frameworks.

## 3 Results and discussion

### 3.1 Individual models

**Figure 4:**
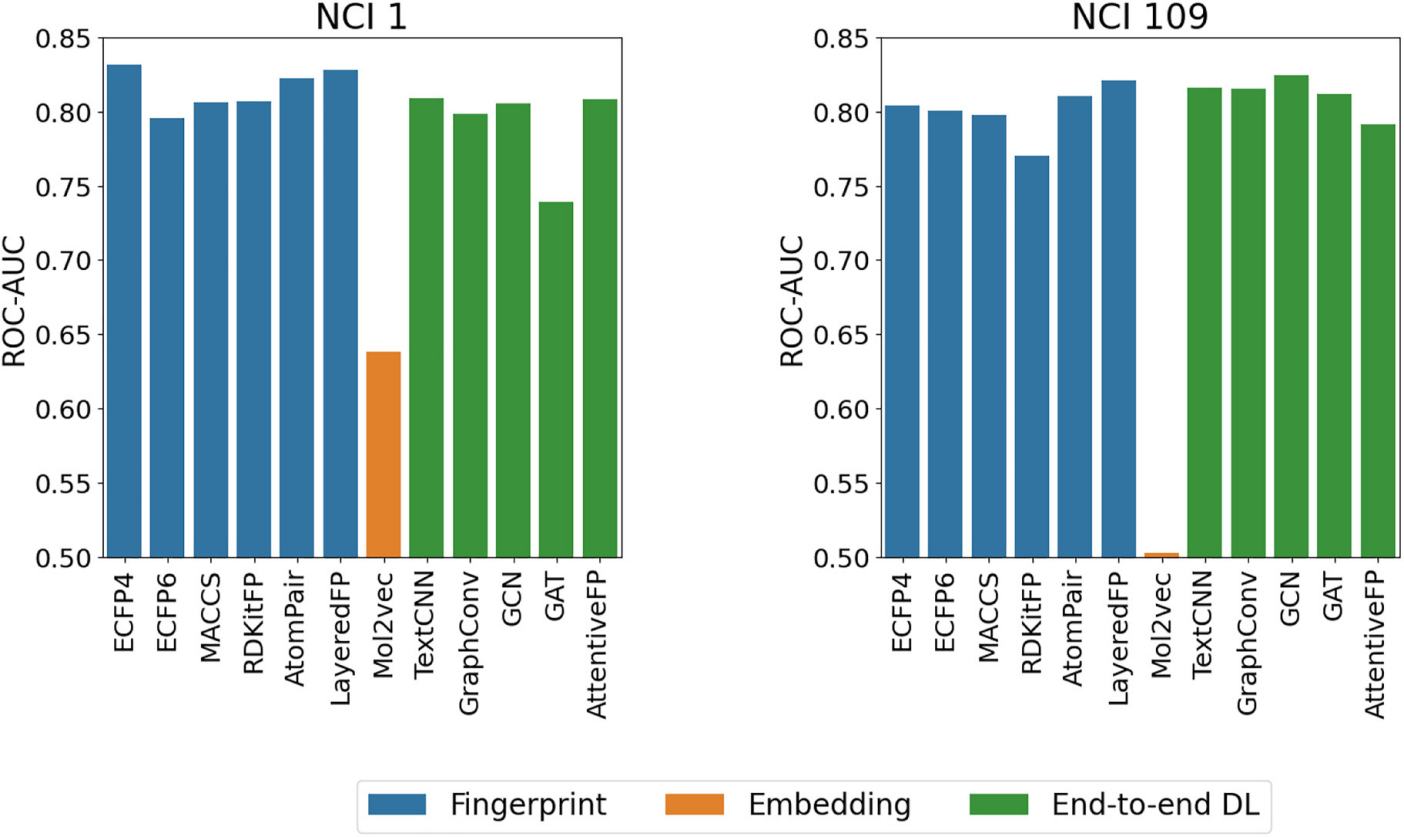
Performance (ROC-AUC) of different deep learning models on the NCI 1 and NCI 109 classification tasks. Higher scores mean better performance.

**Figure 5:**
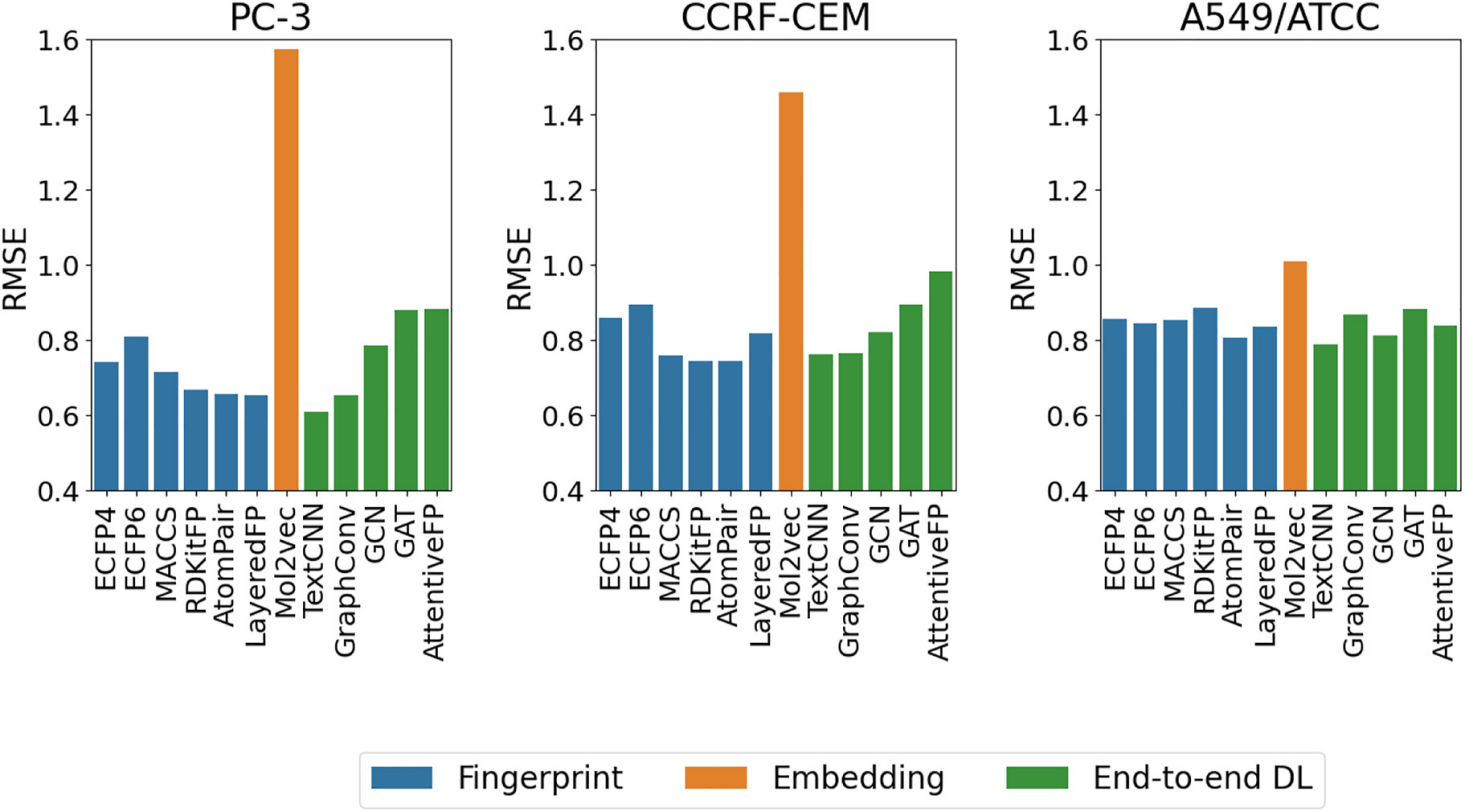
Performance (RMSE) of different deep learning models on the PC-3, CCRF-CEM and NCI-60 A549 regression tasks. Lower scores mean better performance.

In this section, we report and discuss the performance of 12 DL algorithms benchmarked on 5 drug response datasets of variable size and with different output variables. Figure 4 reports the results for classification tasks, with model performance quantified using the area under the receiver operating characteristic curve (ROC-AUC). For regression problems, model performance scores are reported in Figure 5, using the root mean squared error (RMSE) values. The full results tables and plots for additional scoring metrics are available from https://github.com/BioSystemsUM/DeepMol/tree/pacbb21/pacbb21_paper/results.

ECFP4 fingerprints outperformed other fingerprints and end-to-end DL models on the NCI 1 classification task, having achieved a ROC-AUC score of 0.83. The LayeredFP model achieved a very similar ROC-AUC score (also 0.83, when rounded), while the best end-to-end DL model (TextCNN) reached a score of 0.81. On the NCI 109 dataset, the GCN algorithm ranked first in terms of performance, but other methods such as LayeredFP, TextCNN and GraphConv were not far behind (Figure 4). With the exception of the LayeredFP model, fingerprint-based methods generally performed worse than most of the end-to-end DL models on this dataset.

On the PC-3 dataset, the TextCNN model achieved the lowest RMSE (0.61), followed by the LayeredFP model and the GraphConv model, both with an RMSE of 0.65 (Figure 5). Other GNNs did not perform as well as GraphConv, having been surpassed by most of the fingerprint-based models. In the CCRF-CEM regression task, several fingerprint-based models (AtomPair, RDKitFP and MACCS) outperformed the best end-to-end DL model, which was once again the TextCNN model (RMSE = 0.76) (Figure 5). On the larger A549 dataset (Figure 5), TextCNN was the best model (RMSE = 0.79), followed by AtomPair and GCN which both reached an RMSE value of 0.81. The increase in dataset size did not benefit all of the end-to-end DL models, however.

In general, performance scores were usually similar between many of the models. This finding is in agreement with the MoleculeNet benchmarking study, which was also unable to find clear differences between models when benchmarking on smaller datasets (less than 3000 compounds) [11]. The best type of compound representation method seems to depend on the dataset itself, even when the prediction tasks are similar. Other factors, such as the particular data split that was used or the limited number of hyperparameter combinations that were explored using randomized search, could also have influenced the results. Surprisingly, results were consistently worse when using Mol2vec embeddings, which were generated using a model that had been pre-trained on a dataset with over 19 million molecules [6]. The Mol2vec models performed poorly on the training sets as well, indicating that these models were underfitting. Although the original Mol2vec study does not mention the need for scaling the embeddings before using them as inputs to ML models, some of the values in the embeddings are outside the ideal range (between 0 and 1) for DL models. Scaling the embeddings prior to learning might be necessary to improve learning. In addition, dataset-specific fine-tuning of the embedding model might also improve the performance of FCNNs trained on these embeddings.

End-to-end DL models performed as well as, and at times even surpassed, models trained on pre-computed features. TextCNN models, in particular, ranked highly across all datasets.

GNNs also performed relatively well on some of the prediction tasks. This is contrary to what would be expected given the limited number of compounds and the fact that these models were not pre-trained on larger chemical datasets beforehand. However, more complex GNNs with attention mechanisms (GAT and AttentiveFP) did not always perform as well as other end-to-end methods on the datasets that were used in this study, indicating that applying self-attention to the molecular graph nodes does not seem to be particularly advantageous in this case.

Regarding molecular fingerprints, LayeredFP consistently performed well across all datasets, and models trained using atom pair fingerprints also performed relatively well, both outperforming the more popular ECFP fingerprints in 4 out of 5 datasets. LayeredFPs are able to encode information about larger subsets of the molecular graphs than ECFPs, capturing more information on the global structure of the molecules. AtomPair fingerprints also encode more global features since all pairs of atoms are taken into account. The results suggest that global molecular features might be important for the prediction of drug sensitivity. Therefore, these less well-known fingerprints might be interesting alternatives to some of the more commonly used options, at least for drug response prediction tasks.

Despite the use of regularization methods, most models still had a tendency to overfit. In the future, an early stopping mechanism similar to the Keras EarlyStopping callback could be implemented for all models to try to mitigate this.

### 3.2 Ensemble models versus individual models

For each prediction task, the best individual models were also compared to two different ensembles: an 11-model ensemble comprising all models except Mol2Vec models, and an ensemble comprising only the 5 best individual models for a specific task. Results for the classification tasks and the regression tasks are provided in Tables 2 and 3, respectively.

**Table 2:** Ensemble results for classification tasks.

Task	Model	ROC-AUC	Accuracy	PRC-AUC	Precision	Recall
	ECFP4	0.831	0.831	0.870	0.826	0.831
NCI 1	11-Model ensemble	0.862	0.862	0.898	0.881	0.831
	5-Model ensemble	0.851	0.852	0.888	0.866	0.825
	GCN	0.824	0.824	0.864	0.809	0.842
NCI 109	11-Model ensemble	0.856	0.856	0.894	0.878	0.822
	5-Model ensemble	0.845	0.845	0.882	0.847	0.838

**Table 3:** Ensemble results for regression tasks.

Task	Model	RMSE	Pearson	R^2^	Spearman
	TextCNN	0.607	0.806	0.641	0.773
PC-3	11-Model ensemble	0.581	0.831	0.671	0.797
	5-Model ensemble	0.555	0.837	0.700	0.811
	AtomPair	0.742	0.798	0.629	0.761
CCRF-CEM	11-Model ensemble	0.706	0.840	0.664	0.817
	5-Model ensemble	0.645	0.849	0.720	0.825
	TextCNN	0.787	0.667	0.421	0.593
A549/ATCC	11-Model ensemble	0.741	0.715	0.487	0.641
	5-Model ensemble	0.736	0.711	0.494	0.637

Using the 11-model ensembles improved performance scores across all prediction tasks relative to the best individual models for nearly all of the scoring metrics considered. The 5-model ensembles also outperformed single models on all tasks, and performed better than the larger ensembles on the regression tasks, having achieved lower RMSE scores. The improvement in performance, when using ensembles instead of single models, is likely due to the fact that different representations capture different molecular features. Therefore, combining several drug representations is a strategy that should be considered when developing drug response prediction models in the future.

### 3.3 Feature importance

**Figure 6:**
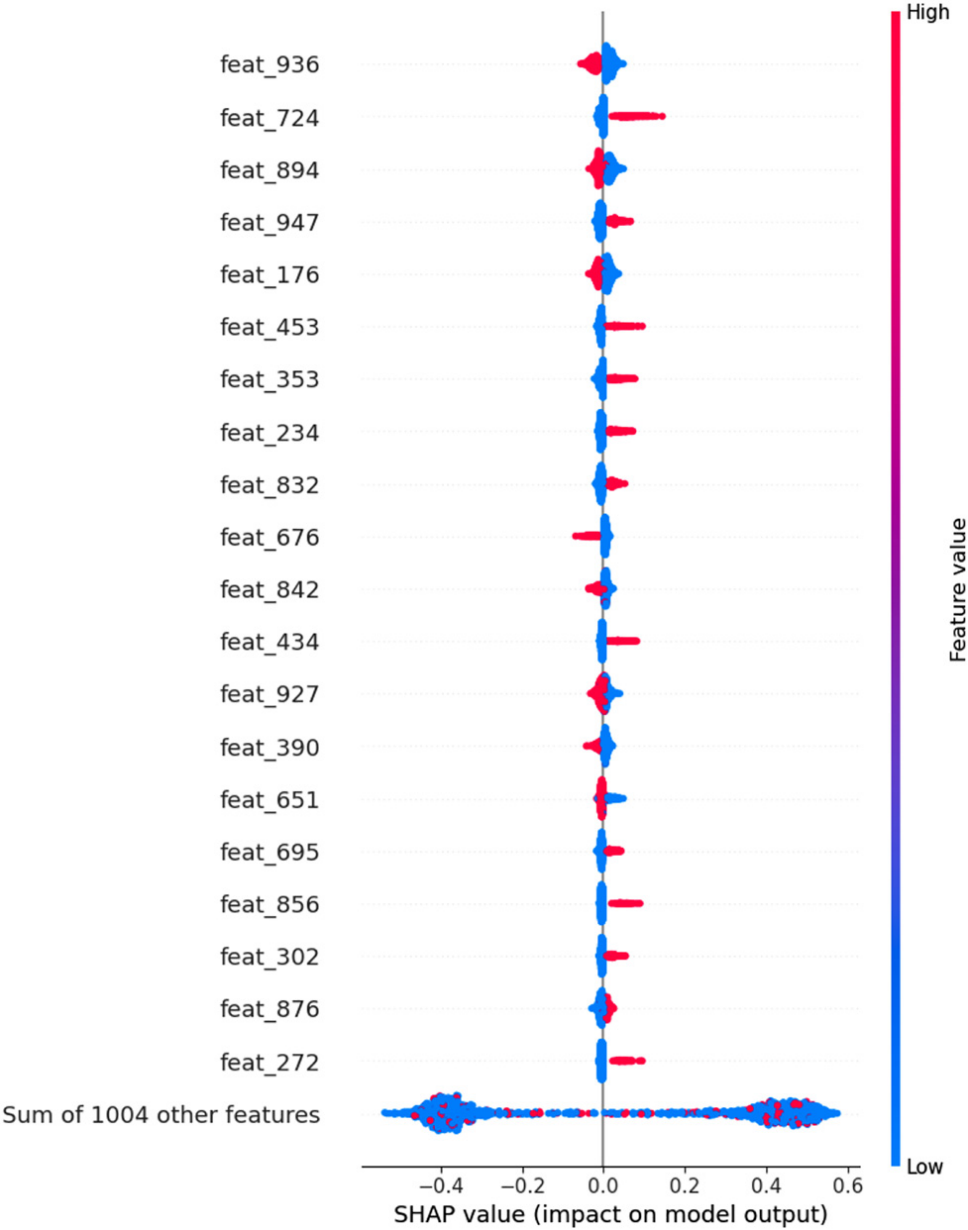
Global feature importance for the ECFP4 model trained on the NCI 1 dataset. Only the 20 most important features are shown, and features are ordered according to their importance. Each point represents a sample in the test set, and the color of the points represents the value of a particular feature for that sample.

**Figure 7:**
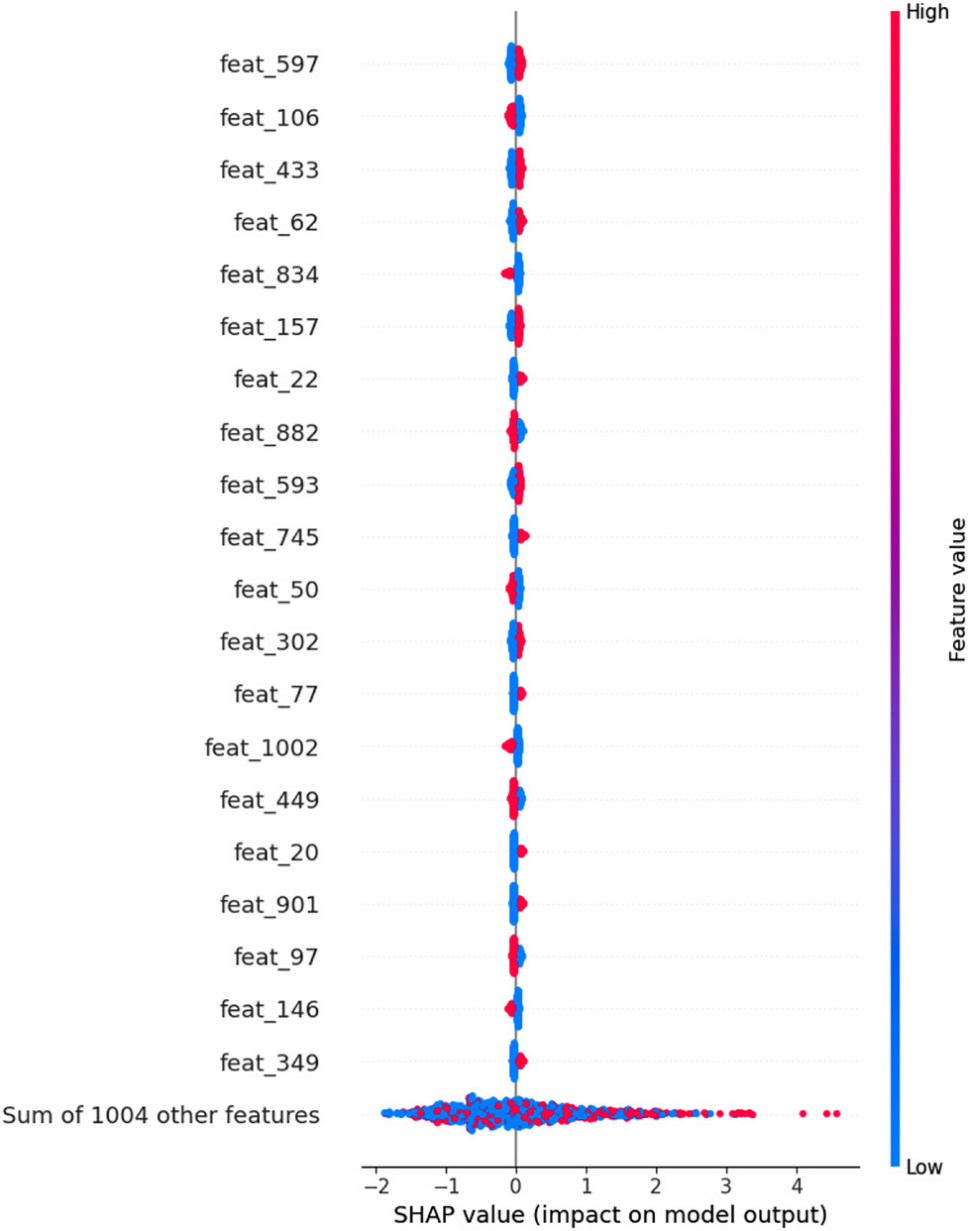
Global feature importance for the AtomPair model trained on the CCRF-CEM dataset. Only the 20 most important features are shown, and features are ordered according to their importance. Each point represents a sample in the test set, and the color of the points represents the value of a particular feature for that sample.

SHAP values were used to determine which features were the most important for the test set predictions. The top 20 most important features (calculated for the test sets) for the best individual models for the NCI 1 classification task (ECFP4 model) and the CCRF-CEM regression task (AtomPair model) are shown in Figures 6 and 7, respectively. Features with greater absolute SHAP values will be the most important, and global feature importance can be determined by averaging the absolute SHAP values across all samples in the dataset. Through the use of color, these plots also show how the value of a given feature impacts the model prediction.

For NCI 1, the SHAP values can be used to determine which fingerprint bits generally lead the model to predict whether the cell line will be sensitive to a given compound or not when the bits are set to 1. For example, ECFP4 bits 724, 947, 453, 353, 324, 832, 434, 695, 856, 302, and 272 have a positive effect on the model output, indicating that the presence of these fragments in a compound is associated with drug sensitivity. Likewise, in the CCRF-CEM task, fingerprint bits that have higher SHAP values are compound fragments that, when present, contribute to increase the pIC_50_ values. This indicates that the sensitivity of the CCRF-CEM cell to treatment is greater when these substructures are present in the screened compounds.

**Figure 8:**
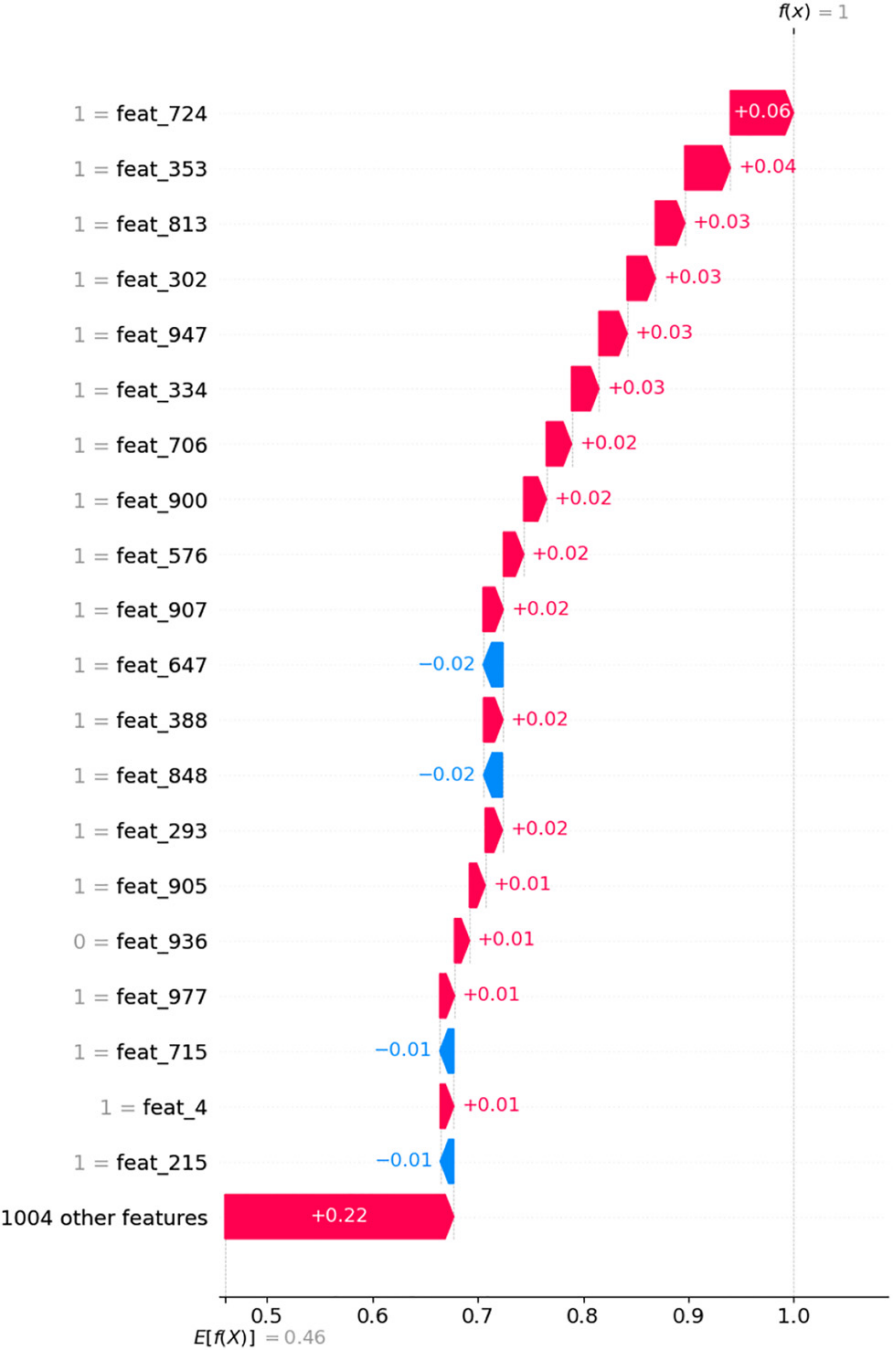
SHAP explanation of the prediction made by the ECFP4 model for sample 537 from the NCI 1 test set, represented as a waterfall plot. Features are ordered by their impact on the model output. Only the top 20 features that contribute the most to the prediction are shown.

SHAP values can also be analyzed at the sample level, allowing to explain how specific features affect a single prediction. Figure 8 shows how each feature contributes to increase or decrease the model prediction from a base value of 0.46 for sample 537 from the NCI 1 test set, which had been correctly predicted as “sensitive” (class label = 1) by the ECFP4 model. Fingerprint bits that are set to 1 and move the predicted value towards 1, are features present in the compound that explain the sensitivity of the cell line used in the NCI 1 assay towards this compound.

**Figure 9:**
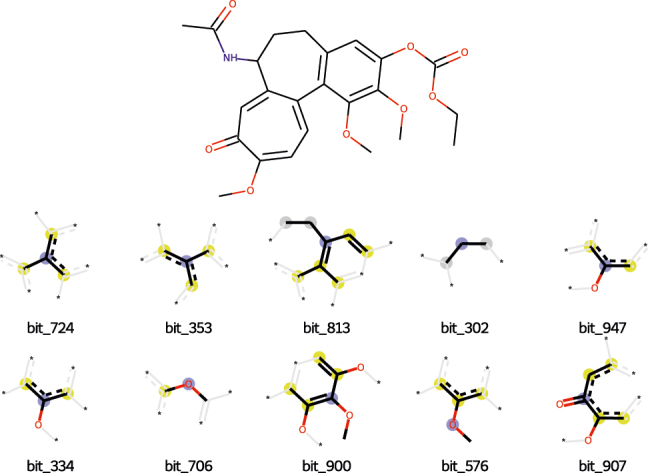
Molecular fragments corresponding to the ten ECFP4 bits that contribute the most towards the prediction for sample 537 from the NCI 1 test set.

Figure 9 depicts the top ten ECFP4 bits and the corresponding substructures that most influenced the model prediction for sample 537. The compound in question is (7-Acetamido-1,2,10-trimethoxy-9-oxo-6,7-dihydro-5H-benzo[a]heptalen-3-yl) ethyl carbonate, a colchicine analog. Colchicine and its analogs bind to tubulin [36]. The top 10 most important ECFP4 bits identified for sample 537 correspond to substructures that have been identified as essential for the bioactivity of colchicine binding site inhibitors [36, 37]. Bit 900 corresponds to the trimethoxyphenyl ring that is essential for interaction with the binding site [36]. Bits 576 and 706 also represent parts of the trimethoxyphenyl moiety, and are centered on atoms that are part of the methoxy groups where drug-protein interactions are more likely to occur [36]. Bits 813, 724 and 334 are centered on atoms belonging to the phenyl ring that is part of the trimethoxyphenyl group as well, which has been shown to be essential for maintaining the geometry of the molecule required at the binding site [36]. Bit 907 is centered on a keto group where an important drug-tubulin interaction can occur in the form of a hydrogen bond [36, 37], and bit 947 is centered on a carbon atom that is linked to a methoxy group, which also might potentially be involved in drug-protein interactions [36]. Bit 302 is more difficult to interpret, but it appears to be representing part of the central 7-membered ring. These results indicate that the DL model was capable of learning which molecular features are the most relevant for predicting drug response in this particular case.

It would also be possible to determine which AtomPair fingerprint bits are most predictive of drug sensitivity for specific samples in the CCRF-CEM test set using the calculated SHAP values. However, due to the use of a hashed version of AtomPair fingerprints with a fixed length, obtaining more information on the specific fragments that are associated with each fingerprint bit is not possible, making these fingerprints more difficult to interpret than ECFP4 fingerprints.

SHAP values were only calculated for the NCI 1 and CCRF-CEM tasks because we were unable to apply SHAP directly to TextCNN (best type of model for the PC-3 and A549/ATCC datasets) and GCN (best model on the NCI 109 task). More explanation methods, including graph-specific methods such as GNNExplainer [38], should be explored the future.

Although SHAP helped to identify important features and explain how individual features affect the output of some of the DL models developed in this study, it is still difficult to explain how the interactions between multiple drug features influence drug response. The use of alternative ML methods that produce human-readable models, such as Decision Trees or other rule induction algorithms, or relational learning algorithms (e.g. inductive logic programming), may be preferable when a greater degree of interpretability is required.

## 4 Conclusions

Most compound representation benchmarking studies are focused on molecular property prediction or drug-target interaction prediction tasks. The aim of this study was to determine which types of representations perform best for the specific problem of drug sensitivity prediction in cancer cell lines. Both traditional molecular fingerprints and DL-based representation learning methods were compared. Our findings show that most compound representations perform similarly. Nevertheless, this comparison of methods allowed the identification of representation strategies that consistently performed well across different drug response datasets. Some end-to-end DL models were capable of performing as well as or even better than traditional fingerprint-based models even on smaller datasets. Additionally, less well-known molecular fingerprints may be interesting alternatives to some of the more popular types of molecular fingerprints. The effect of combining multiple compound representation methods into ensembles of models was also evaluated in this study. The results of this analysis show that this strategy led to improved predictive performance. Finally, this study has also shown that SHAP can be used to increase the interpretability of fingerprint-based DL models. These findings can help guide the development of new DL-based drug response prediction models trained on screening data from other screening projects.

The chemoinformatics software package that was used to carry out these analyses is under constant development and it is currently at a pre-release version. New models and features will be added in the future.

## References

[1] Ali M, Aittokallio T (2019). Machine learning and feature selection for drug response prediction in precision oncology applications. Biophys Rev.

[2] Adam G, Rampášek L, Safikhani Z, Smirnov P, Haibe-Kains B, Goldenberg A (2020). Machine learning approaches to drug response prediction: challenges and recent progress. npj Precis Oncol.

[3] Cereto-Massagué A, Ojeda MJ, Valls C, Mulero M, Garcia-Vallvé S, Pujadas G (2015). Molecular fingerprint similarity search in virtual screening. Methods.

[4] Duvenaud D, Maclaurin D, Aguilera-Iparraguirre J, Gómez-Bombarelli R, Hirzel T, Aspuru-Guzik A (2015). Convolutional networks on graphs for learning molecular fingerprints. J Chem Inf Model.

[5] Xiong Z, Wang D, Liu X, Zhong F, Wan X, Li X (2020). Pushing the boundaries of molecular representation for drug discovery with the graph attention mechanism. J Med Chem.

[6] Jaeger S, Fulle S, Turk S (2018). Mol2vec: unsupervised machine learning approach with chemical intuition. J Chem Inf Model.

[7] Mayr A, Klambauer G, Unterthiner T, Steijaert M, Wegner JK, Ceulemans H (2018). Large-scale comparison of machine learning methods for drug target prediction on ChEMBL. Chem Sci.

[8] Jiang D, Wu Z, Hsieh CY, Chen G, Liao B, Wang Z (2021). Could graph neural networks learn better molecular representation for drug discovery? A comparison study of descriptor-based and graph-based models. J Cheminf.

[9] Hop P, Allgood B, Yu J (2018). Geometric deep learning autonomously learns chemical features that outperform those engineered by domain experts. Mol Pharm.

[10] Zagidullin B, Wang Z, Guan Y, Pitkänen E, Tang J (2021). Comparative analysis of molecular fingerprints in prediction of drug combination effects. Briefings Bioinf.

[11] Wu Z, Ramsundar B, Feinberg EN, Gomes J, Geniesse C, Pappu AS (2018). MoleculeNet: a benchmark for molecular machine learning. Chem Sci.

[12] Pappu A, Paige B (2020). Making graph neural networks worth it for low-data molecular machine learning. Machine learning for molecules workshop @ NeurIPS 2020.

[13] Yang K, Swanson K, Jin W, Coley C, Eiden P, Gao H (2019). Analyzing learned molecular representations for property prediction. J Chem Inf Model.

[14] Pan S, Wu J, Zhu X, Long G, Zhang C (2015). Finding the best not the most: regularized loss minimization subgraph selection for graph classification. Pattern Recogn.

[15] Cortés-Ciriano I, Bender A (2019). KekuleScope: prediction of cancer cell line sensitivity and compound potency using convolutional neural networks trained on compound images. J Cheminf.

[16] Mendez D, Gaulton A, Bento AP, Chambers J, De Veij M, Félix E (2019). ChEMBL: towards direct deposition of bioassay data. Nucleic Acids Res.

[17] Yang W, Soares J, Greninger P, Edelman EJ, Lightfoot H, Forbes S (2013). Genomics of Drug Sensitivity in Cancer (GDSC): a resource for therapeutic biomarker discovery in cancer cells. Nucleic Acids Res.

[18] Seashore-Ludlow B, Rees MG, Cheah JH, Coko M, Price EV, Coletti ME (2015). Harnessing connectivity in a large-scale small-molecule sensitivity dataset. Cancer Discov.

[19] Bento AP, Hersey A, Félix E, Landrum G, Gaulton A, Atkinson F (2020). An open source chemical structure curation pipeline using RDKit. J Cheminf.

[20] Rogers D, Hahn M (2010). Extended-connectivity fingerprints. J Chem Inf Model.

[21] Morgan HL (1965). The generation of a unique machine description for chemical structures-A technique developed at chemical abstracts service. J Chem Doc.

[22] Durant JL, Leland BA, Henry DR, Nourse JG (2002). Reoptimization of MDL keys for use in drug discovery. J Chem Inf Comput Sci.

[23] Carhart RE, Smith DH, Venkataraghavan R (1985). Atom pairs as molecular features in structure-activity studies: definition and applications. J Chem Inf Comput Sci.

[24] Landrum G (2006). RDKit: Open-source cheminformatics.

[25] Kim Y (2014). Convolutional neural networks for sentence classification. Proceedings of the 2014 conference on empirical methods in natural language processing (EMNLP).

[26] Ramsundar B, Eastman P, Walters P, Pande V, Leswing K, Wu Z (2019). Deep Learning for the Life Sciences: Applying Deep Learning to Genomics, Microscopy, Drug Discovery, and More.

[27] Kipf TN, Welling M (2017). Semi-supervised classification with graph convolutional networks. 5th International conference on learning representations, ICLR 2017, Toulon, France, April 24-26, 2017, conference track proceedings.

[28] Velickovic P, Cucurull G, Casanova A, Romero A, Liò P, Bengio Y (2018). Graph attention networks. 6th International conference on learning representations, ICLR 2018, Vancouver, BC, Canada, April 30–May 3, 2018, conference track proceedings.

[29] Kingma DP, Ba J (2014). Adam: a method for stochastic optimization. Proceedings of the 3rd international conference on learning representations.

[30] Srivastava N, Hinton G, Krizhevsky A, Sutskever I, Salakhutdinov R (2014). Dropout: a simple way to prevent neural networks from overfitting. J Mach Learn Res.

[31] Lundberg SM, Lee SI, Guyon I, Luxburg UV, Bengio S, Wallach H, Fergus R, Vishwanathan S (2017). A unified approach to interpreting model predictions. Advances in neural information rocessing systems 30.

[32] Shrikumar A, Greenside P, Kundaje A (2017). Learning important features through propagating activation differences. Proceedings of the 34th international conference on machine learning-volume 70.

[33] Abadi M, Barham P, Chen J, Chen Z, Davis A, Dean J (2016). Tensorflow: a system for large-scale machine learning. Proceedings of the 12th USENIX symposium on operating systems design and implementation.

[34] Chollet F (2015). Keras.

[35] Pedregosa F, Varoquaux G, Gramfort A, Michel V, Thirion B, Grisel O (2012). Scikit-learn: machine learning in Python. J Mach Learn Res.

[36] McLoughlin EC, O’Boyle NM (2020). Colchicine-binding site inhibitors from chemistry to clinic: a review. Pharmaceuticals.

[37] Nguyen TL, McGrath C, Hermone AR, Burnett JC, Zaharevitz DW, Day BW (2005). A common pharmacophore for a diverse set of colchicine site inhibitors using a structure-based approach. J Med Chem.

[38] Ying R, Bourgeois D, You J, Zitnik M, Leskovec J (2019). Gnnexplainer: generating explanations for graph neural networks. Adv Neural Inf Process Syst.

